# Automated highly multiplex detection system for respiratory pathogens in canines

**DOI:** 10.3389/fvets.2025.1722097

**Published:** 2026-01-09

**Authors:** Wing Shing Wong, Xie Lin, Parker Y. L. Tsang, Johnson Y. N. Lau, Lok-Ting Lau

**Affiliations:** 1 Emerging Viral Diagnostics (HK) Limited, Hong Kong, Hong Kong SAR, China; 2 Faculty of Science, Hong Kong Baptist University, Hong Kong, Hong Kong SAR, China; 3 Institute for Innovation and Translation, Hong Kong Baptist University, Kowloon Tong, Kowloon, Hong Kong SAR, China; 4 Department of Applied Biology and Chemical Technology, The Hong Kong Polytechnic University, Hong Kong, Hong Kong SAR, China; 5 Wu Jieh Yee Institute of Translational Chinese Medicine Research, Hong Kong Baptist University, Hong Kong, Hong Kong SAR, China; 6 Department of Industrial and Systems Engineering, The Hong Kong Polytechnic University, Hong Kong, Hong Kong SAR, China

**Keywords:** multiplex PCR, CIRD, canine respiratory pathogens, automation, point-of-care diagnostics

## Abstract

**Introduction:**

Canine infectious respiratory diseases (CIRDs) are prevalent causes of respiratory illnesses in dogs. Clinical signs are non-specific, including coughing, rhinorrhea, and fever, making it challenging for veterinarians, especially at the onset of symptoms, to identify the causative pathogens based on clinical presentation alone. On the other hand, early and accurate diagnosis is crucial for preventing progression to severe complications such as pneumonia and widespread outbreaks. The ability to differentiate between viral and bacterial etiologies can guide appropriate treatment and medication directions, such as avoiding misuse of antibiotics. Therefore, this study aimed to develop a novel multiple molecular assay suitable for an automated detection system using a nested polymerase chain reaction (PCR) method. The assay covers 14 common canine respiratory pathogens using 15 gene targets, including canine influenza virus (H3N2, H3N8, and H1N1), canine distemper virus, canine parainfluenza virus, canine herpesvirus, pseudorabies virus, rabies virus, canine adenovirus (types 1 and 2), canine coronavirus, *Mycoplasma canis*, *Bordetella bronchiseptica*, and *Streptococcus equi subsp. zooepidemicus*.

**Methods:**

Their primers and probes in the assay were first developed according to the nested PCR protocol and designed parameters required in the automated system. This developed assay has then been rigorously validated.

**Results and discussion:**

The assay developed has demonstrated high analytical sensitivity and specificity. The results obtained from the automated system are comparable to those from conventional laboratory procedures. The assay has shown possibility in rapidly detecting multiple pathogens in canines, and its utility can potentially be extended beyond companion animals to other mammalian species as well. Its application can enhance infection surveillance in animal populations and potentially mitigate zoonotic transmission risks.

## 1 Introduction

Canine infectious respiratory disease (CIRD) is contagious among dogs. This can be caused by a single or combination of various viral and bacterial pathogens (1). In this category, common pathogens include canine coronavirus (CCoV), which was first detected in the UK in 2003 and has spread widely worldwide (2). *Bordetella bronchiseptica* (*B. bronchiseptica*) is one of the most prevalent bacterial pathogens and is often presented in coinfection with other pathogens, which can exacerbate the symptoms (3). Canine adenovirus-type 2 (CAdV-2) (4) and canine parainfluenza virus (CPIV) (5) typically result in mild respiratory symptoms but can occasionally develop into more severe illness. Canine distemper virus (CDV) is a highly contagious virus that can lead to systemic disease and rapid spread in kennels (6). Canine herpesvirus-1 (CHV-1) primarily can affect the reproductive system (7). Canine influenza viruses (CIVs), particularly the H3N8 and H3N2 subtypes, are serious threats (8, 9). CIRD is a global health concern having various geographical distributions. For example, the bacteria, *B. bronchiseptica* and the virus CPIV, presented in 29.4% of household dogs, exhibited respiratory infection symptoms in Japan (10). On the other hand, CCoV was popular in the southeastern United States, which showed a positivity rate of 14% in CIRD cases (11). In environments with crowded canine population density, such as kennels and animal shelters, the spread of CIRD can be fast through increased chance of airborne and contact transmission (12, 13). In U.S. shelters, *B. bronchiseptica* and *Mycoplasma canis* (*M. canis*) were the most common pathogens, accounting for 19.5 and 51.4% of all cases, respectively (14). The early onset of an outbreak can be unnoticeable because the infected dogs can start shedding pathogens even before clinical symptoms manifest, causing asymptomatic transmission in their population (14). As pathogens continue to mutate, the impact can be enormous (15, 16). The risk is not only confined to canines but also humans because CIRD pathogens can potentially evolve and become cross-species transmissions, posing a devastating threat to public health (17, 18).

In the management of CIRD, diagnosis can act as a critical frontline defense that enables timely and accurate medication, preventing disease progression into severe pneumonia (15). Furthermore, diagnosis can lead to prompt isolation and intervention, breaking chains of transmission at the early onset of pandemics. Conventionally, diagnosing canine respiratory pathogens relies on medical history, clinical symptoms, and laboratory tests (19). Diagnosis based on appearing symptoms is unreliable because pathogens in such a group can cause the same or very similar clinical signs, such as typical coughing, sneezing, and rhinorrhea. It also cannot determine coinfection caused by random combinations of more than one pathogen. Methods used in laboratory testing include bacterial culture, enzyme-linked immunosorbent assay (ELISA), and molecular-based amplification methods, such as reverse transcription polymerase chain reaction (RT-PCR) (20). Bacterial culture is considered to be the “gold standard” for identifying bacterial pathogens, but it requires days to complete and is not applicable to viral pathogens (21). ELISA is a common serological detection method and can have results typically within an hour. Its sensitivity is rather low; therefore, pathogens can be detected after illness progression but not at its early onset. Therefore, testing at multiple time points is recommended to confirm infection (22).

Molecular-based detections, particularly RT-PCR, are increasingly adopted due to their accuracy and rapidness (23). Through thermal cycles consisting of denaturation, annealing, and extension, the sequences of ribonucleic acid (RNA) and deoxyribonucleic acid (DNA) of pathogens presented in testing samples can specifically be amplified and identified (24). Its sensitivity and specificity depend on the primer’s design (25). The addition of fluorescent detection probes can further enhance specificity and enable real-time quantification (26–28). In application to CIRD, multiplex PCR detection incorporating multiple sets of primers and probes is even more suitable than singleplex PCR, as it can cover more pathogens (29–31). Previous studies have shown significant usefulness of using multiplex assay (32–34) for coinfection diagnosis, epidemiological surveillance, and environmental monitoring for laboratory animals. Increasing the number of pathogens in a panel is desired but challenging (35, 36). It is because when all primer pairs and probes are pooled in a single PCR reaction, specificity can be compromised due to interference and non-specific binding among the primers themselves and with target templates. Furthermore, unifying the annealing temperature (Tm) of all the primers is hard for fitting into a single thermal profile. Careful primer probe design is necessary; however, it is a complex process due to primer dimer formation, non-specific binding, and mismatches between primers and templates (30). Non-optimized primer sets will lead to false-positive results, reduced detection limits, and unpredictable amplification results. In regard to instrumentation, multiplexing capability is limited by the number of optical channels equipped in a fluorescence PCR cycler, i.e., less than 8 targets (37).

To realize highly multiplex PCR, nested PCR was therefore proposed (38–40). It splits a PCR process into two stages of amplification. The first stage can be a highly multiplex PCR process. The amplicons produced from the first-stage amplification were then diluted and aliquoted into respective tubes containing a new master mix with a pair of primers and a probe specific to a pathogen for undergoing the second-stage singleplex PCR amplification. This nested approach can recover both specificity and sensitivity. However, performing nested PCR requires extra pipetting work and can increase the risk of contamination of a molecular laboratory due to manipulation of amplicons. These elevate demand in operation, and this method is hard to adopt routinely in the laboratory.

In order to facilitate convenient use of the nested PCR protocol, the Avalon Automated Multiplex System (AAMST) has been developed, realizing detection of multiple respiratory pathogens in humans (38). It is a fully automated complete molecular testing protocol, including extraction of nucleic acids, a nested PCR, and an optical system for fluorescent signal acquisition. The system consists of a microfluidic cartridge (AAMC), a main analyzer (AAMA), which is the driver house for the cartridges, and control software (AAMS), which is the user interface and result viewer for the system. AAMST has demonstrated excellent consistency in performance and ease of operation, providing a complete solution for scenarios requiring multiple detections. This system can potentially be extended to many other groups of diseases, including the potential use for CIRD. This extended system can realize laboratory-free diagnosis of CIRD, especially in primary care facilities, shelters, and rural communities. Such an advantage is significant for surveillance and control of infectious diseases and early diagnosis and prevention of disease outbreaks (41–43).

In this study, an attempt is made to develop a novel automated multiplex detection panel for CIRD operated on the AAMST system. Primers and probes were first designed following the parameters required for system integration. Unique plasmids were constructed as positive controls to verify analytical performance. The detection performance of the system is then further validated using inactivated pathogens as reference materials in the sample input. The developing panel covering 14 pathogens with 15 gene targets in concern of CIRD is listed in Table 1. It can subtype CIV and differentiate between viruses and bacteria. Their abbreviations, gene targets, and detecting regions are also given.

**Table 1:** Pathogens and corresponding gene targets covered in the nested PCR assay development.

Pathogens	Abbreviation	Gene target	GenBank accession number
Virus			
Canine influenza virus	CIV	Matrix	PP576969.1
Canine influenza A virus (H3N2)	H3N2 CIV	Hemagglutinin (HA)	CY044261.1
Canine influenza A virus (H3N8)	H3N8 CIV	Hemagglutinin (HA)	CY032381.1
Canine influenza A virus (H1N1)	H1N1 CIV	Hemagglutinin (HA)	MW204471.1
Canine distemper virus	CDV	Nucleoprotein	AF378705.1
		Matrix protein	
Canine parainfluenza virus	CPIV	Nucleocapsid protein	MW273368.1
Canine herpesvirus	CHV-1	Glycoprotein B (gB)	MW353129.1
		UL36	
Pseudorabies virus	PRV	Glycoprotein D (gd)	PV157261.1
		Fusion protein (UL22)	
Rabies virus	RABV	Nucleoprotein	PP965371.1
		Nucleoprotein	
Canine adenovirus type 2	CAdV-2	Hexon protein	MN402910.1
Canine adenovirus type 1	CAdV-1	Hexon gene	OR466089.1
Canine coronavirus	CCoV	Nucleocapsid protein (N)	JF682842.1
Bacteria			
*Bordetella bronchiseptica*	*B. bronchiseptica*	Fimbrial protein	CP043114.1
*Mycoplasma canis*	*M. canis*	Elongation factor Tu (tuf)	CP132191.1
*Streptococcus equi subsp. zooepidemicus*	SEZ	manB	CP071146.1
		Hypothetical cytosolic protein	

## 2 Materials and method

### 2.1 Design of primers and probes

**Figure 1:**
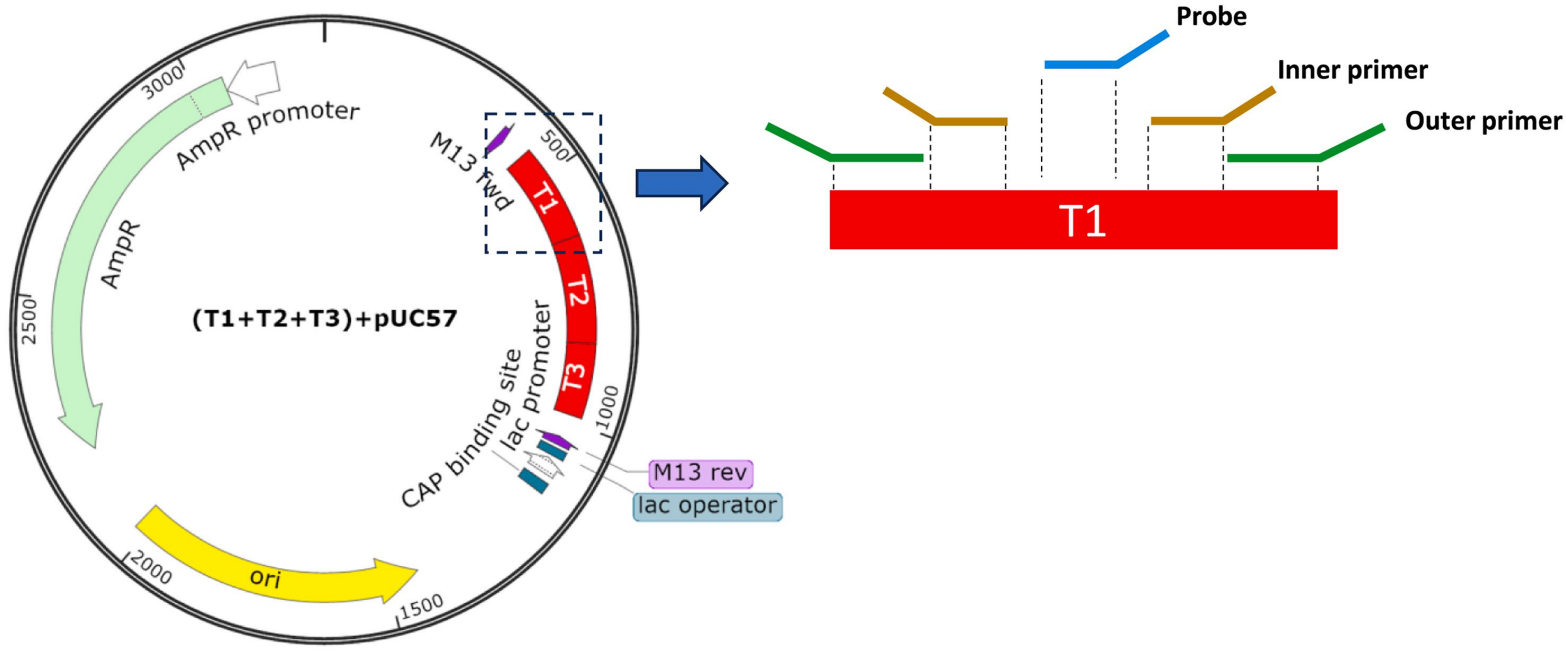
Illustration of the plasmid designed in this study. Three target sequences, i.e., T1, T2, and T3, indicated in the red region with ~550 bp, were inserted into the plasmids and served as positive control materials for assay verification. The region of T1 was further zoomed in for illustrating positions of the outer primer, inner primer, and probe in the nested PCR system.

Their specific genomic sequences of the pathogen targets were found from the National Center for Biotechnology Information (NCBI, https://www.ncbi.nlm.nih.gov/). Primers and probes were designed in their conserved regions as shown in Table 1. In reference to the genomic sequences, the outer primers were used to produce longer amplicons in the first-stage multiplex PCR amplification. The amplicons were then received by the binding of the inner primers and probes in the second-stage singleplex real-time PCR. Their positions are illustrated in the zoom-in region of Figure 1, where T1 is a fragment of genomic sequence to be incorporated into the plasmid. Several sets of primers for each gene target shall be designed for assessment and further selection. In the first-stage amplification, outer primers are designed for high melting temperature (Tm) close to 70 °C to achieve high specificity in the multiplexing PCR. Therefore, their primer sequences were constructed using longer lengths and higher GC% ratios, whereas, in the second-stage amplification, the inner primers were designed to be shorter in length and hence have a relatively lower melting temperatures close to 60 °C. Fluorescent probes are located at the target sequence between a pair of inner primers. During the design phase, all the sequences of primers and probes shall avoid the formation of stable dimers or hairpin structures. Non-specific primer binding to other targets shall also be rejected for minimizing undesirable amplifications. Parameters used in the design phase are summarized in Table 2.

**Table 2:** Design parameters of the primer probes in this study.

Primer/Probe type	Parameter type	Parameter range mean (range)
Outer primers	Length of amplicon	200 (143–284) bp
	Primer length	33 (20–41) bp
	GC%	50 (38–75) %
	Melting temperature (Tm)	71 (70–72) °C
Inner Primers	Length of amplicon	111 (78–182) bp
	Primer length	22 (16–29) bp
	GC%	47 (26–69) %
	Melting temperature (Tm)	62 (61–63) °C
Probe	Probe length	29 (19–39) bp
	Probe GC% range	50 (39–71) %
	Melting temperature (Tm)	69 (68–72) °C

### 2.2 Design of the plasmid positive control

In order to verify the design of primers and probes in terms of their amplification efficiency and analytical performance, unique plasmids were constructed to serve as positive templates. A plasmid is a closed circular DNA molecule with a relatively higher molecular weight and longer length than synthesized oligonucleotides and therefore is possible to cater to multiple target sequences. As illustrated in Figure 1, the pUC57 plasmid cloning vector (44) is used. The plasmid has approximately 3.3 kbp in length and can be inserted with three target fragments, namely, T1, T2, and T3, respectively, as indicated in the red region (~550 bp). Each target fragment follows the sequences of targeting pathogens and allows complementary binding with designed primers and probes to designated positions during amplification processes. Following the designs, production of these plasmids was carried out by Sangon Bioengineering (Shanghai) Co., Ltd. In brief, plasmid quantity was first increased by expression using *Escherichia coli* competent cells and was then purified using chromatography. Their target sequences were confirmed by sequencing. The concentration of the plasmids was measured by optical absorbance using a NanoDrop spectrophotometer (Thermo Fisher Scientific, Waltham, MA, United States). The measured values, A260, were used to convert into copy numbers per milliliter (copies/mL) using the formula copies/mL = (A260 (ng/*μ*L) × 10^−6^ × 6.02 × 10^23^)/(DNA length × 650). The plasmids were serially diluted to a range between 2.8 × 10^6^ and 2.8 × 10^2^ copies/mL and stored at -20 °C before use. Nine plasmids with different designs, including a total of 27 target sequences, were used during the development.

### 2.3 Nucleic acid extraction by laboratory procedure

Input plasmid templates or samples (300 *μ*L) were first mixed with 150 *μ*L of lysis buffer for 15 s. 600 *μ*L of binding buffer was then added and mixed well for 15 s. The sample mixture was then centrifuged at 8000 rpm for 1 min through an RNase-free column (TianGen). The column was washed with 600 *μ*L of wash buffer with centrifugation at 8000 rpm for 1 min. The column was added to 100 *μ*L of nuclease-free water and incubated for 1 min at room temperature. Eluent was collected by centrifuging the column at 14000 rpm for 1 min.

### 2.4 Conditions and analytical linearity of nested PCR

In the first-stage multiplex RT-PCR, 5 μL of sample template was mixed with 6.25 *μ*L of enzyme master mix. All outer primers, both forward and reverse primers, were mixed together and added into the enzyme master mix with a final concentration of 0.07 *μ*M. Nuclease-free water was used to top up the reaction volume to 25 *μ*L. The RT-PCR reaction included reverse transcription at 50 °C for 5 min. After deactivation of RT at 95 °C for 20 s, PCR was followed by thermal cycling of 95 °C for 6 s and 65 °C for 35 s for 26 cycles. The product was then diluted 20 times with nuclease-free water before starting the second-stage singleplex PCR. In the second-stage singleplex PCR, single pairs (forward and reverse) of inner primers (0.5 *μ*M), FAM probe (0.2 *μ*M), and master mix consisting of PCR enzyme (5 *μ*L) and 0.2 *μ*M Rox dye were prepared in individual tubes, respectively, for each panel pathogen. A measure of 3.6 *μ*L of diluted amplicon quantitative PCR (qPCR) was then added into each tube and taken to a cycler (QuantStudio 7 Flex Real-Time PCR System, Thermo Fisher) for performing qPCR. The thermal cycling profile was set to be 95 °C for 1 s and 55 °C for 45 s for 45 cycles. For analytical evaluation, a linearity plot of threshold cycle (CT) values was plotted against concentrations of input template using the quantitative plasmid templates, as described in section 2.2, with serial 10-fold dilution from a concentration of 2.8 × 10^6^ to 2.8 × 10^2^ copies/mL.

### 2.5 Experiment using AAMST

**Figure 2:**
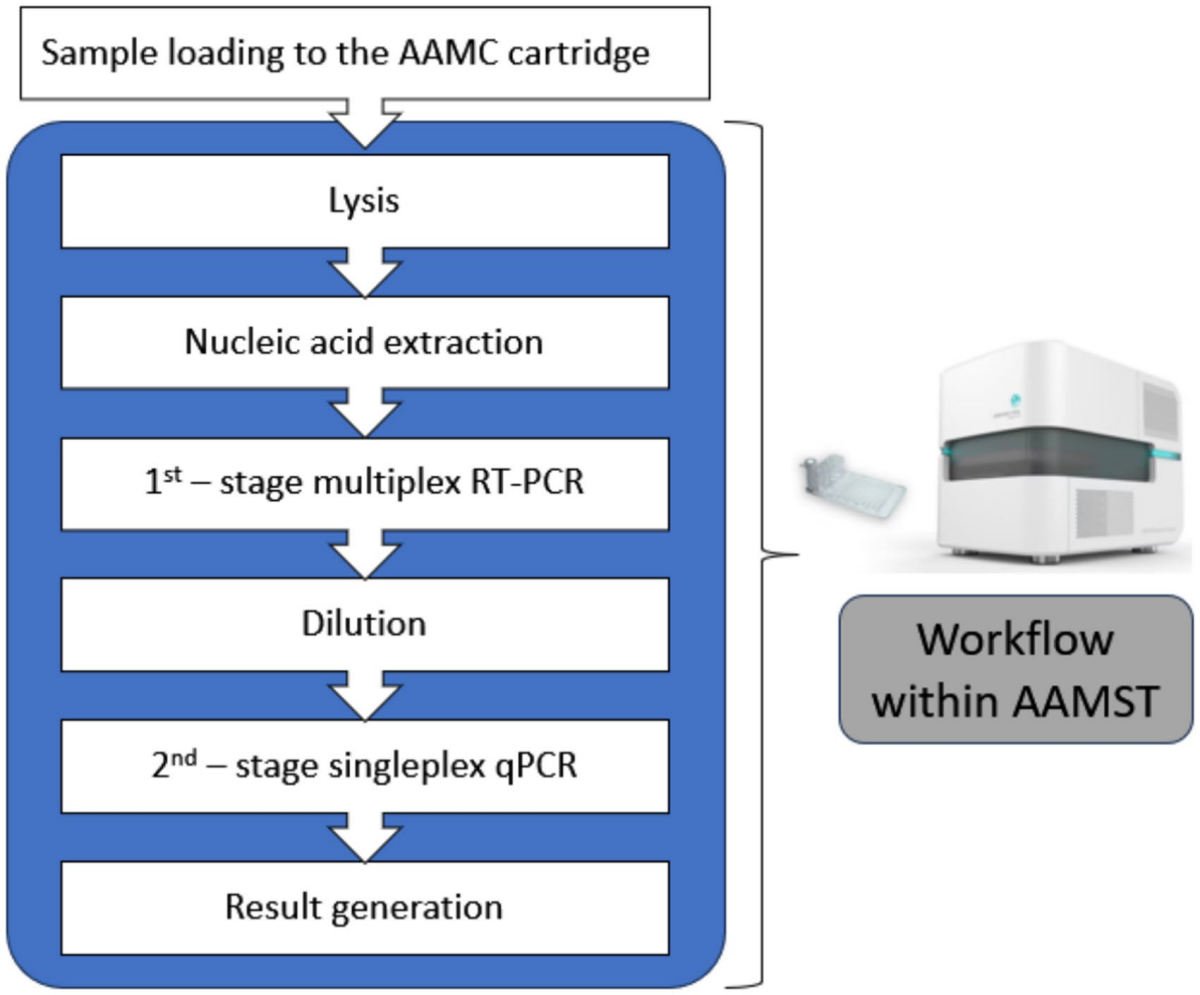
Illustration of workflow performed in the AAMST system.

Details in the description and the workflow operated in the AAMST were referred to Tsang et al. (38). In the experiment, a 300 *μ*L sample was loaded into the AAMC cartridge. This was then sent into the AAMA analyzer. The testing procedure can be started with a click on the button of AAMS software. Automation runs through the defined biochemistry protocol, the same as those described in sections 2.3 and 2.4, without further human operation and intervention. In brief, with the illustration of Figure 2, steps run in the system included cell lysis, nucleic acid extraction (i.e., purification and isolation), nested PCR, signal acquisition, data analysis, and result reporting. The basic principle is that the AAMC has incorporated designed primers and probes and reagent sets during production. It also has the well-defined microfluidic network, consisting of fluidic paths, control valves, pumps, and reaction chambers in which an array of 120 lightbulbs serves as reaction chambers for the second-stage qPCR. The AAMA analyzer, equipped with electronic, mechanical, and electrothermal units, actuates the components on the AAMC cartridge, such as pumps and valves for controlling fluidic movement. Its temperature control units provide a temperature ramping profile to fulfill the PCR cycles. The optical system was equipped for emitting narrow-band light for excitation and acquisition of fluorescent signals. The entire process took approximately 90 min for result generation.

### 2.6 Assay validation for AAMST

The assay was validated using inactivated pathogens purchased from Shengcheng Beina Chuanglian, BNCC, which has complete genomic sequences of the target clinical pathogens. Their original concentrations and traceability are listed in Table 3. It is noted that the concentration has been determined by quantitative PCR and their sequences have been verified by gene sequencing at the manufacturer. The inactivated samples were used as sample inputs to the AAMST and were stored in a -80 °C freezer before use.

**Table 3:** List of the reference material used for assay verification.

^a^Reference materials	^b^Concentration [copies/ml]	Traceability
CIV-H1N1	6.8 × 10^7^	ATCCVR-1469
CDV	2.1 × 10^5^	ATCCVR-1587
CHV-1	2.3 × 10^7^	ATCCVR-552
CAdV-2	4.3 × 10^5^	ATCCVR-800
CAdV-1	8.3 × 10^5^	ATCCVR-293
CPIV	6.5 × 10^7^	ATCCVR-1573
*B. bronchiseptica*	1.3 × 10^7^	ATCC-19395
SEZ	2.5 × 10^5^	ATCC-43079

a Sequences were verified by sequencing.

b Their original concentrations were determined by quantitative PCR.

## 3 Result

### 3.1 Verification of the designed primers and probes in the multiplex nested PCR

**Figure 3:**
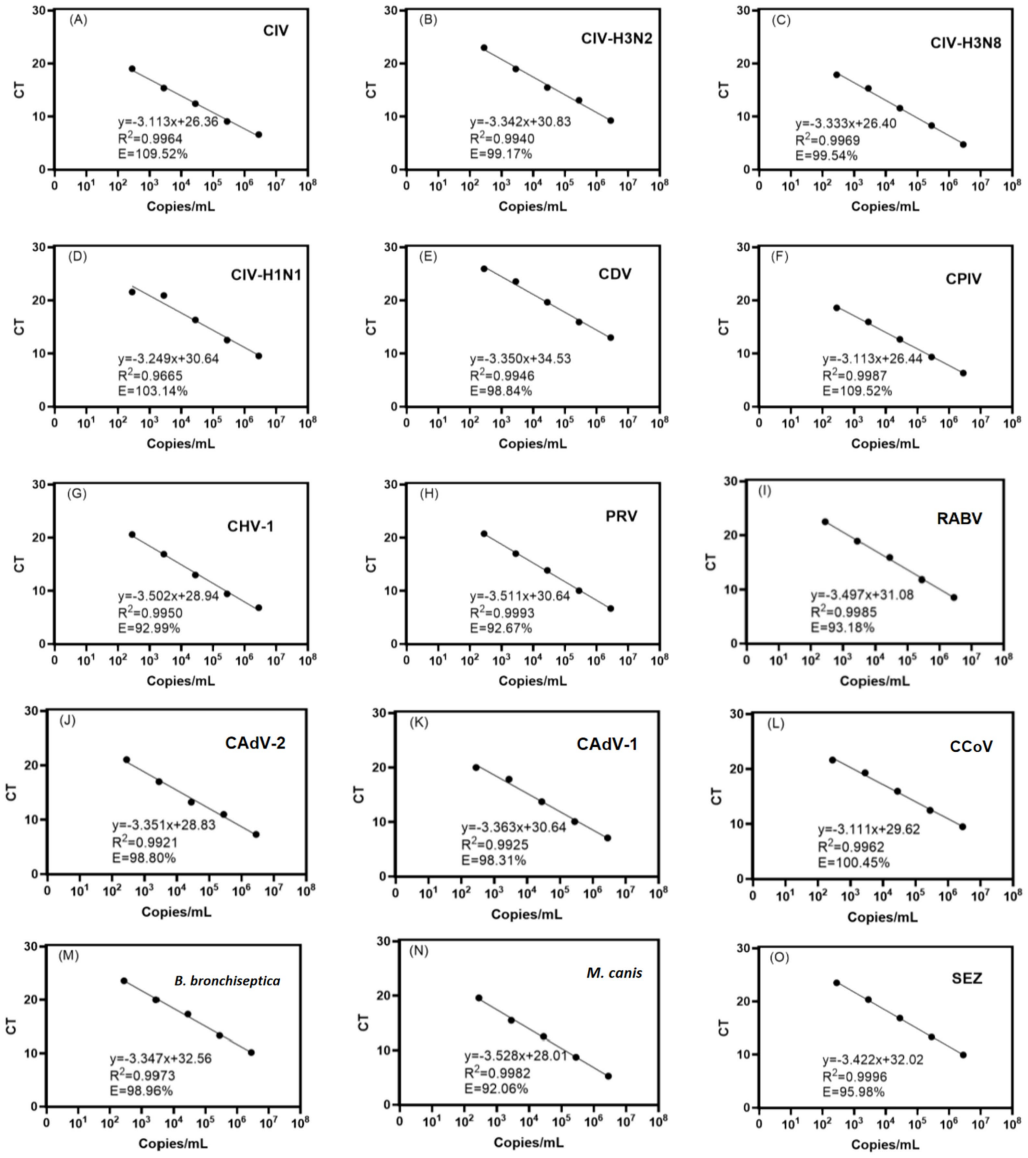
Plot of cycle threshold (CT) against serial dilutions of target concentrations using the designed primer probes in the proposed multiplex nested PCR amplification. Linear trendlines and their equations are shown. Satisfactory linearity, R-squared values (R^2^), and amplification efficiency (E) are obtained for all the targets **(A–O)** covered in the proposed panel assay.

The primers and probes designed in this study were first verified using benchtop procedures and equipment before being implemented into the AAMC cartridges. This verification ensures that they are compatible with the thermal profiles as required in the proposed nested PCR protocol. It is desired to achieve satisfactory amplification efficiency, linearity, and detection limit. The plasmid positive controls containing the target sequences with concentrations ranging from 2.8 × 10^2^ to 10^6^ copies/mL were used as input templates with serial ten-fold dilutions. The templates first underwent an extraction process for purification and isolation of nucleic acids and then underwent first-stage multiplex amplification with outer primers, dilution, and the second-stage singleplex amplification with inner primers and probes, sequentially. The results are shown in Figure 3, in which Figures 3A–O plot the threshold cycle (CT) values against concentrations of each input template. Each data point is an average value of triplicates with a deviation of < 1 CT. As shown in the figures, for all 15 detection targets, typical linear standard curves were obtained. Satisfactory linearity and amplification efficiency have been demonstrated. All respective PCR amplification curves were inspected (data not shown), and all illustrated standard sharps, having clear plateaus above baseline. It is noted that, in the design process, multiple sets of primers and probes were designed. Those sets that failed to achieve satisfactory amplification curves, such as low plateau levels and high CT, were eliminated from the assay. Qualified primer and probe sets were applied to the AAMC production.

### 3.2 Analytical sensitivity of AAMST

**Figure 4:**
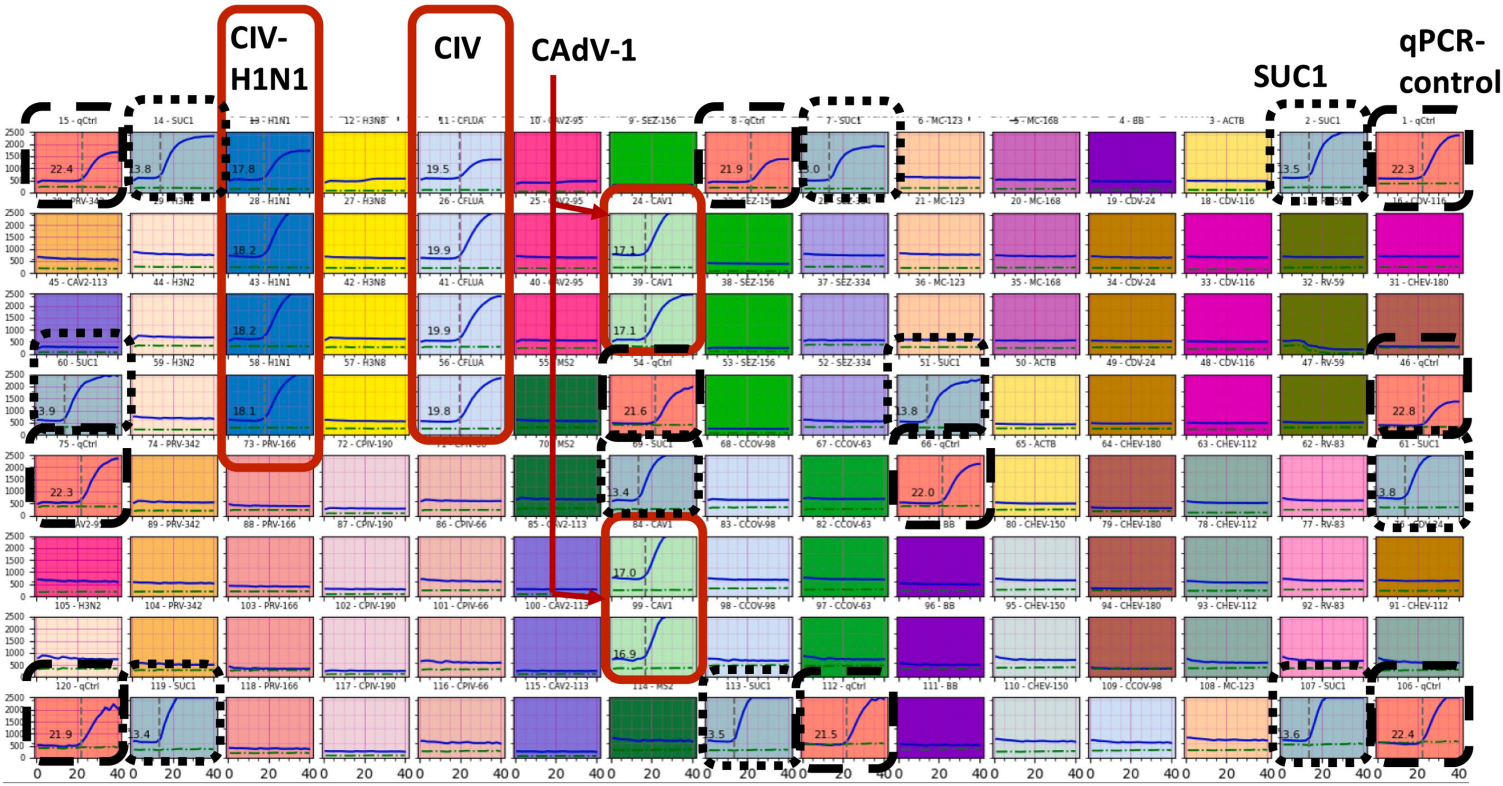
PCR amplification curves acquired from each reaction chamber are all shown in an array format. Valid amplification curves according to the input templates were embraced in red solid-line boxes, whereas those for in-process controls were in two different dashed-line boxes. CIV, CIV-H1N1, and CAdV-1 were correctly detected. The detection was robust in all the replicates and in-process controls (SUC1 and qPCR control).

For verifying compatibility of the developed assay with the AAMST system, the plasmids were used as input templates for loading into the AAMC cartridges with the selected primers and probes incorporated. The cartridges were then processed in the system. Typical results of the amplification curves are shown in Figure 4, in which 800 copies/mL of the plasmid constructed with three targets, CIV, CIV-H1N1, and CAdV-1, were used as input templates. As shown in the figure, the curves were consistently and specifically obtained from the corresponding reaction chambers where their complementary primers were located, whereas the curves were absent from other chambers. It is noted that *Schizosaccharomyces pombe* RNA (SUC1) and qPCR controls were used for in-process controls of first and second-stage amplifications, respectively, ensuring a proper system run (38). Afterward, various concentrations of templates, 80 copies/mL, 800 copies/mL, and 4,000 copies/mL, were used to test the lowest detectable concentrations. Each concentration has triplicate repeats. Only those concentrations that detected valid CT values in all triplicates by the system are reported. As shown in Table 4, the lowest detectable concentrations [limit of detection (LOD)] can reach as low as 4,000 copies/mL or 4 copies/μL for both *M. canis* and CCoV and 800 copies/mL or 0.8 copies/μL for all other 13 targets. These values generated by this automated multiplexing system are comparable to the data reported in the literature for relevant canine assays using conventional PCR detections (29, 32, 45–48). It is also noted that during the detection, excellent specificity was observed. This result provided verification of the designed primers and probes and excellent compatibility with AAMST, particularly working well with the thermal profile and automation defined by the system.

**Table 4:** Detectable lowest concentrations from the AAMST incorporating the proposed primers and probes.

Target	LOD (copies/mL)
CIV	8 × 10^2^
CIV-H3N2	8 × 10^2^
CIV-H3N8	8 × 10^2^
CIV-H1N1	8 × 10^2^
CDV	8 × 10^2^
CPIV	8 × 10^2^
CHV-1	8 × 10^2^
PRV	8 × 10^2^
RABV	8 × 10^2^
CAdV-2	8 × 10^2^
CAdV-1	8 × 10^2^
CCoV	4 × 10^3^
*B. bronchiseptica*	8 × 10^2^
*M. canis*	4 × 10^3^
SEZ	8 × 10^2^

### 3.3 Verification of the canine assay with AAMST using inactivated pathogens

The proposed automated assay is further verified by reference materials using commercial inactivated pathogens, as shown in Table 3 for their information. They were a relevant model used to imitate clinical samples for evaluating feasibility and usability. Various dilutions of the reference materials, as well as combinations of pathogens, were applied in the input sample individually: SEZ, CAdV-2, CAdV-1, CHV-1, CDV, *B. bronchiseptica*, as well as, collectively, a mixture of CIV-H1N1, CPIV, CHV-1, CAdV-2, CAdV-1, *B. bronchiseptica,* and SEZ. As shown in Table 5, for the qualitative results, “positive” was defined when valid CT values, within the brackets of Table 5, are detected from the corresponding pathogens. The CT values can also be used to estimate their quantitative amounts. It is noted that the CT values depend on the concentration of input materials. The numbers in the brackets reflected appropriately with various dilutions prepared prior to the runs. For the pathogens absent in the input materials, no amplification curves were shown, and hence CT values are undetermined from the corresponding PCR chambers, as represented by blank cells in Table 5. In the detection of the sample with a mixture of pathogens, the results are all consistent with the known inputs. This result has verified the capacity of the system to perform a multiplex detection. It has also demonstrated the use of multiplex detection with agreement of specificity for subtyping influenza, i.e., positive to CIV and CIV-H1N1, differentiating CAdV-1 and CAdV-2, and detecting pools of pathogen mixtures, i.e., viruses (CIV-H1N1, CPIV, CHV-1, CAdV-2, and CAdV-1) and bacteria (*B. bronchiseptica* and SEZ) in the sample. This is highly relevant to co-infection cases.

**Table 5:** Detection results of inactivated pathogens using the developed assay in AAMST.

Assay targets input materials	CIV	CIV -H3N2	CIV -H3N8	CIV-H1N1	CDV	CPIV	CHV-1	PRV	RABV	CAdV-2	CAdV-1	CCoV	*B. bronchiseptica*	*M. canis*	SEZ
SEZ															Positive (7.10)
CAdV-2										Positive (3.30)					
CAdV-1											Positive (3.13)				
CHV-1							Positive (3.30)								
CDV					Positive (13.11)										
*B. bronchiseptica*													Positive (32.05)		
CIV-H1N1 CPIV CHV-1 CAdV-2 CAdV-1 *B. bronchiseptica* SEZ	Positive (17.33)			Positive (16.50)		Positive (20.87)	Positive (9.87)			Positive (17.03)	Positive (15.53)		Positive (8.27)		Positive (15.80)

Remark: the numbers in brackets show the CT values obtained from the tests.

## 4 Discussion

An assay development and analytical verification have been reported for an automated multiplex detection of 14 pathogens with 15 gene targets associated with canine respiratory diseases. The assay performance in terms of limit of detection (LOD) achieved in this study ranged between 8 × 10^2^ and 4 × 10^3^ copies/mL, highly comparable to other data reported in literature using laboratory procedures. Excellent specificity is also obtained, which showed absence in non-specific amplification. This is attributed to the use of the nested PCR protocol. In the nested PCR, the first-round multiplex PCR might sacrifice specificity, leading to amplicons with unintended sequences due to non-specific binding of primers. However, this can be tolerated because the amplicons were then verified again during the second-round singleplex PCR, where new sets of primers and additional probes were used. As a result, LOD and specificity for multiplex amplification can both be achieved.

During the development, it is worth pointing out that verifying designed primers before integrating them onto the cartridge is significantly critical. Primers are not only targeting conserved regions of the pathogens but also have to be compatible with the restrictive thermal profiles and avoid unwanted interactions with other primers and sequence fragments. Failures can be avoided by performing careful Tm calculation and gene alignment at the design phase, but it is often unreliable. Experimental verification by observing their resultant amplification curves and CT values is essential; Particularly, this can be confirmed by the linearity plots as shown in Figure 3. After this verification, the subsequent integration to the cartridge is straightforward. Further primer screening and selection can be dependent on performance with reference materials and clinical samples. In future studies, the assay performance has to be further confirmed with large-scale evaluation with a significant number of clinical samples to define statistical clinical sensitivity and specificity. Moreover, the effects of the sample matrix in canines on the assay performance will need to be addressed. Nevertheless, it is assumed that the effects would be similar to human samples. The sample matrix in humans was well-accepted by the assay in the AAMST system (38, 49).

In the treatment for coinfection, combinations of antiviral and antibiotic drugs can be used. However, the drugs are highly species- and pathogen-specific. For treating infection of a virus, antiviral drugs, such as ribavirin and interferon-*α*, can be considered (50, 51), whereas, for treating infection of bacteria, antibiotics, such as doxycycline, can be applied (52). These are two distinct medication approaches. One type of drug is efficacious for one species but not another. The drugs have potential adverse effects on health. A proper diagnosis should precede the application of drugs. This helps prevent unnecessary or inappropriate medication and potential harm. Furthermore, antibiotics can be prescribed precisely, and hence this can alleviate the increasingly recognized antimicrobial resistance. Since coinfection often occurs in canine respiratory diseases, accurate detection of multiple pathogens, such as the one developed in this study, is important to its implementation and useful to define proper clinical treatments.

Automating the protocol in an enclosed cartridge would provide users with a convenient solution in utilizing such a useful molecular method. For comparison, laboratory workflow operating the same protocol takes at least 3 h, including reagent preparation and liquid handling, as well as transfers across instruments. In contrast, the operators in this study can walk away from the system after loading the sample and starting the run. The results can be available in approximately 90 min.

Furthermore, this study has shown the advantage of using multiplex capability to improve detection reliability. CIRD, such as influenza, is known to have a high mutation rate. Increasing gene targets in the assay can ensure sufficient coverage against new mutations and variants in evolving diseases. The detection limit is also varied in detecting regions. For example, in this study, sets of primer probes for both the nucleoprotein gene and matrix protein gene of the CDV are included in the assay. Detection of CDV can still be possible even if a mutation occurs in one gene or the other. In addition to CDV targets, extra sets of primer probes for other targets can also be designed and included in the assay according to the needs. For influenza, identification of its subtype is useful, as particular subtypes might cause more severe illness than others. As a demonstration of subtyping capability, CIV and CIV-H1N1 using inactivated materials were successfully detected. In total, more than 40 detecting targets can be incorporated into the AAMST system. This technology can be a valuable platform. It can offer very comprehensive and versatile detection panels that can be translated into many applications in different fields such as research, clinical study, environmental monitoring, and surveillance.

The proposed design procedure and experimental parameters described in this study are important for the development of new panels operating in the automated AAMST. Clear guidelines can reduce uncertainty and, hence, shorten development time. Following the guideline, the assay can be developed into many derivatives by incorporating new combinations and additions of pathogens, depending on the interests of different specialists, regions, and research topics. New panels are expected to respond closely to fast-changing and emerging infectious diseases. Globalization will inevitably speed up the spread of infectious diseases. It will accelerate risk in disease transmission across species due to increased interactions among human–human, human–animal, and animal–animal (53). It is foreseeable that new pathogens never seen in the past will keep emerging and creating huge negative impacts on society (54). Therefore, the capability and ease of panel expansion are critical to keep up-to-date effectiveness and comprehensiveness of a detecting panel.

## 5 Conclusion

A detection panel using a nested PCR approach that can simultaneously cover 14 common pathogens of canine respiratory diseases has been developed in this study. It has then been migrated successfully into the AAMST system for automating such a complicated protocol by following the design flow and parameters provided. The automated assay has been verified by analytical and inactivated pathogens. Excellent LOD and specificity have been demonstrated. This study not only presents the development of an assay targeting the canine respiratory diseases but also will be a useful reference for developing other multiplex assays of this kind.

## Data Availability

The datasets presented in this study can be found in online repositories. The names of the repository/repositories and accession number(s) can be found in the article/supplementary material.
